# A Mysterious Trigger for Serum Amyloid A (SAA)-Associated Amyloidosis: Insights From an Autopsy Study

**DOI:** 10.7759/cureus.86863

**Published:** 2025-06-27

**Authors:** Hem Sunder Thirumurthy, Aravind Sekar, Vikas Suri, Anupam Lal, Ritambra Nada

**Affiliations:** 1 Histopathology, Postgraduate Institute of Medical Education and Research, Chandigarh, IND; 2 Internal Medicine, Postgraduate Institute of Medical Education and Research, Chandigarh, IND; 3 Radiology, Postgraduate Institute of Medical Education and Research, Chandigarh, IND

**Keywords:** alpha-1 antitrypsin deficiency, autopsy findings, cystic bronchiectasis, hyaline globules, saa-associated amyloidosis

## Abstract

Amyloidosis refers to a heterogeneous group of disorders characterized by the extracellular deposition of insoluble fibrillar proteins, leading to tissue damage and functional impairment. Among these, serum amyloid A (SAA) amyloidosis, previously termed secondary amyloidosis, arises in the context of chronic inflammatory conditions. While common causes include autoimmune diseases and chronic infections, the underlying etiology remains unidentified in a subset of cases. We report a rare case of SAA-associated amyloidosis in a 29-year-old male with a history of childhood-onset cystic bronchiectasis. The patient presented with progressive renal dysfunction and was diagnosed with systemic amyloidosis based on histopathological evaluation. Common autoimmune and infectious etiologies were excluded during life. An autopsy revealed alpha-1 antitrypsin deficiency as the underlying cause of chronic pulmonary inflammation, manifesting as cystic bronchiectasis, which likely triggered sustained elevation of serum amyloid A and subsequent amyloid deposition.

## Introduction

Amyloidosis refers to a group of conditions linked to inherited and inflammatory disorders where fibrillar proteins accumulate and cause tissue damage and impair function. Serum amyloid A (SAA)-associated amyloidosis involves widespread deposits of AA protein and was previously termed secondary amyloidosis due to its association with an underlying inflammatory condition [1]. Chronic inflammatory conditions such as tuberculosis, bronchiectasis, chronic osteomyelitis, rheumatoid arthritis, ankylosing spondylitis, and inflammatory bowel diseases are well-recognized triggers for SAA-associated amyloidosis [2,3]. In this type of amyloidosis, liver cells produce increased levels of SAA in response to cytokines like IL-6 and IL-1 during inflammation, leading to sustained SAA elevation [4]. However, simply elevated SAA production is not enough for amyloid deposition. Two mechanisms have been proposed for amyloid deposition: defective degradation of SAA by monocyte-derived enzymes or inherent structural abnormalities in SAA molecules [5]. Here, we describe a rare cause for SAA-associated amyloidosis in a 29-year-old male with childhood-onset cystic bronchiectasis.

## Case presentation

A 29-year-old gentleman, previously diagnosed with childhood-onset cystic bronchiectasis, presented with complaints of anasarca and progressively worsening shortness of breath (modified Medical Research Council Scale (mMRC II-III)) over six months. Urinalysis revealed significant proteinuria, with a 24-hour urine protein level of 15.4 g/day and a serum creatinine level of 5.64 mg/dL. Ultrasound showed bilateral renal parenchymal disease with kidney sizes within normal limits. A kidney biopsy revealed SAA-associated amyloidosis, and he was initiated on maintenance hemodialysis. Over the next five days, his dyspnea worsened further, reaching mMRC grade III-IV, without associated chest pain, orthopnea, or paroxysmal nocturnal dyspnea. He also experienced five to six episodes of semisolid stools per day without blood for eight to 10 days. There was no history of fever, recurrent oral ulcers, photosensitivity, Raynaud's phenomenon, rash, or focal neurological deficits. He had no personal or family history of diabetes, hypertension, addictions, or similar illnesses.

On examination, he had grade II clubbing, bilateral pedal edema/facial puffiness, bilateral crepitations (more prominent on the left), and no organomegaly. He appeared drowsy and afebrile. His pulse rate was 100/min, blood pressure was 100/60 mmHg, respiratory rate was 26/min, and SpO_2_ was 80% on room air. Respiratory examination revealed bilateral crepitations, while cardiovascular examination showed normal heart sounds. Abdominal examination revealed softness with ascites and audible bowel sounds. CNS examination showed E4V3M3 status without focal neurological deficits. The laboratory findings are summarized in Table 1.

**Table 1:** Summary of laboratory investigations with reference range. ALP: alkaline phosphatase, ALT: alanine aminotransferase, AST: aspartate aminotransferase, CK-NAC: creatine kinase, LDH: lactate dehydrogenase, SSA: Sjögren’s syndrome antigen A, SSB: Sjögren’s syndrome antigen B.

Parameter	Patient value	Reference range
Hemoglobin (g/dL)	7.2	13.5-17.5
Total leukocyte count (/mm³)	10,900	4000-11,000
Platelet count (×10³/µL)	680	150-450
Sodium (Na⁺) (mEq/L)	136	135-145
Potassium (K⁺) (mEq/L)	6.39	3.5-5.0
Urea (mg/dL)	63.2	15-40
Creatinine (mg/dL)	5.64	0.6-1.2
Bilirubin total/conjugated (mg/dL)	0.09/0.04	Total: 0.3-1.2, conjugated: <0.3
AST/ALT/ALP (U/L)	21.7/9.1/107	AST: 10-40/ALT: 7-56/ALP: 44-147
Calcium/phosphate/magnesium (mg/dL)	8.12/6.99/2.3	Ca: 8.5-10.5/PO₄: 2.5-4.5/Mg: 1.6-2.6
Total protein/albumin (g/dL)	5.51/1.30	Total: 6.0-8.3/albumin: 3.5-5.0
LDH (U/L)	209	140-280
CRP (mg/L)	338	<5
Uric acid (mg/dL)	5.3	3.5-7.2 (male)
pH (arterial)	7.239	7.35-7.45
PaCO₂ (mmHg)	47.3	35-45
PaO₂ (mmHg)	58.1	80-100
HCO₃⁻ (mmol/L)	19.7	22-28
Lactate (mmol/L)	1.14	0.5-2.2
Amylase (U/L)	23	30-110
Lipase (U/L)	22.4	13-60
CK-NAC (U/L)	19	30-200
Anti-SSA/Ro and anti-La/SSB	Negative	Negative

Doppler imaging showed thrombosed right axillary and brachial veins. Non-contrast computerized tomography of chest and abdomen suggested small bowel obstruction, dilated jejunal loops, sub-centimetric/enlarged lymph nodes, cystic bronchiectasis, mild right pleural effusion, and centriacinar nodules.

Upon admission, he exhibited hypoxia and received oxygen supplementation. Hyperkalemia was managed medically, followed by hemodialysis. Intravenous fluids and antibiotics were administered before transfer to the emergency medical ward. Extensive investigations were conducted to determine the cause of deterioration. Within the next 24 hours, he required mechanical ventilation. Following intubation, he developed hypotension requiring fluids and inotropes. Antibiotics were upgraded to intravenous colistin and teicoplanin. Despite aggressive management, his condition continued to deteriorate, and he developed paralytic ileus, worsening consciousness, and refractory shock. He eventually succumbed following a cardiac arrest.

Autopsy findings

With due consent from the deceased's relatives, a complete autopsy was performed using a bitemporal midline thoracoabdominal incision. The peritoneal cavity yielded 1500 mL of straw-colored fluid, while the pericardial and pleural cavities were within normal limits.

**Figure 1:**
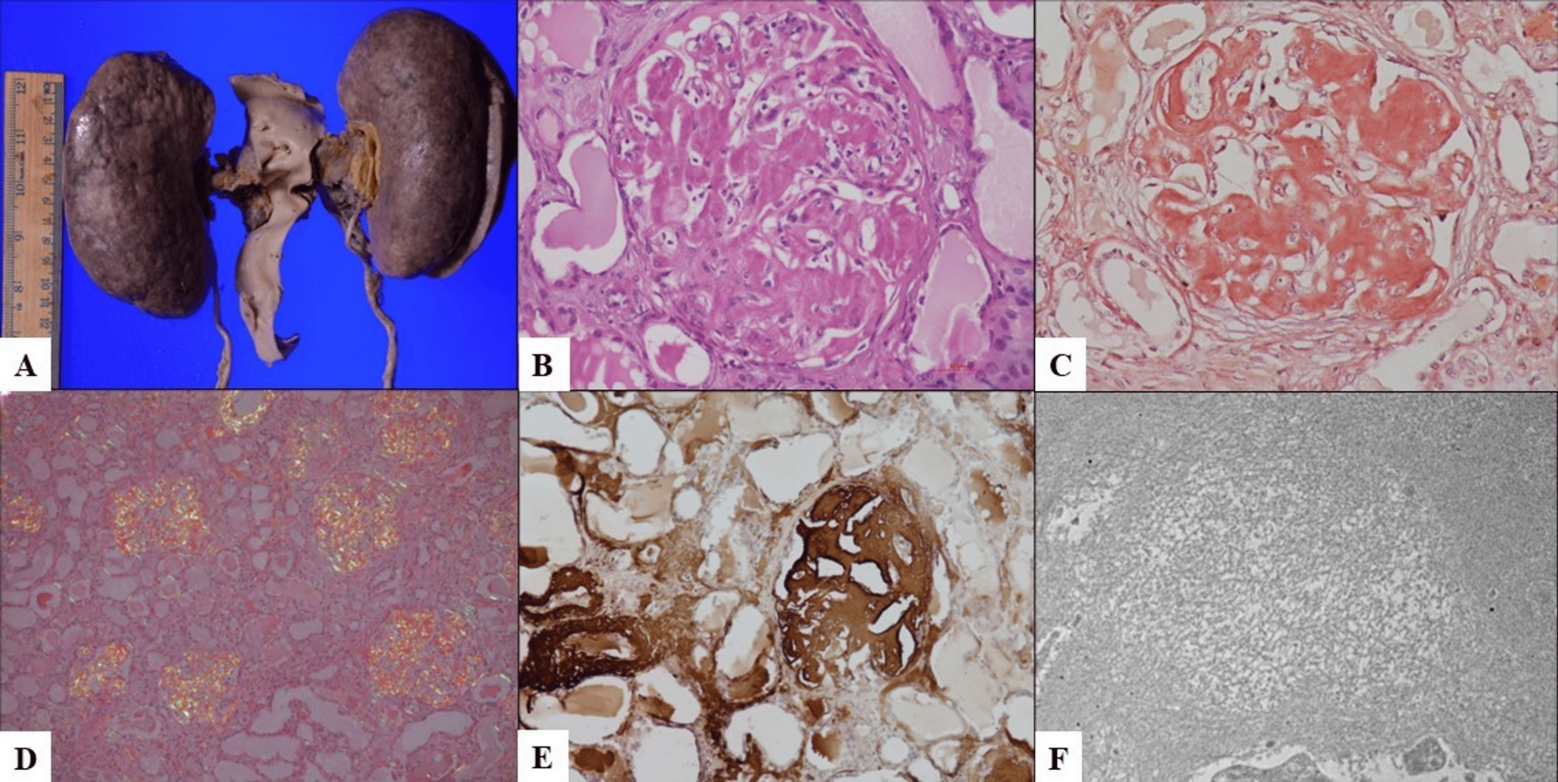
Gross and microscopic findings of kidneys. (A) Gross photograph of the kidneys showing enlargement with nodular capsular surface. (B) Microscopy reveals amorphous, acellular deposits in the mesangium (H&E, 400x). (C) These deposits are congophilic (Congo red, 400x) and show apple-green birefringence under polarized light (D, 100x). (E) Immunohistochemistry reveals strong positivity for serum amyloid A (SAA) protein (400x). (F) Ultrathin electron microscopy shows randomly arranged fibrils measuring 8-12 nm in diameter.

**Figure 2:**
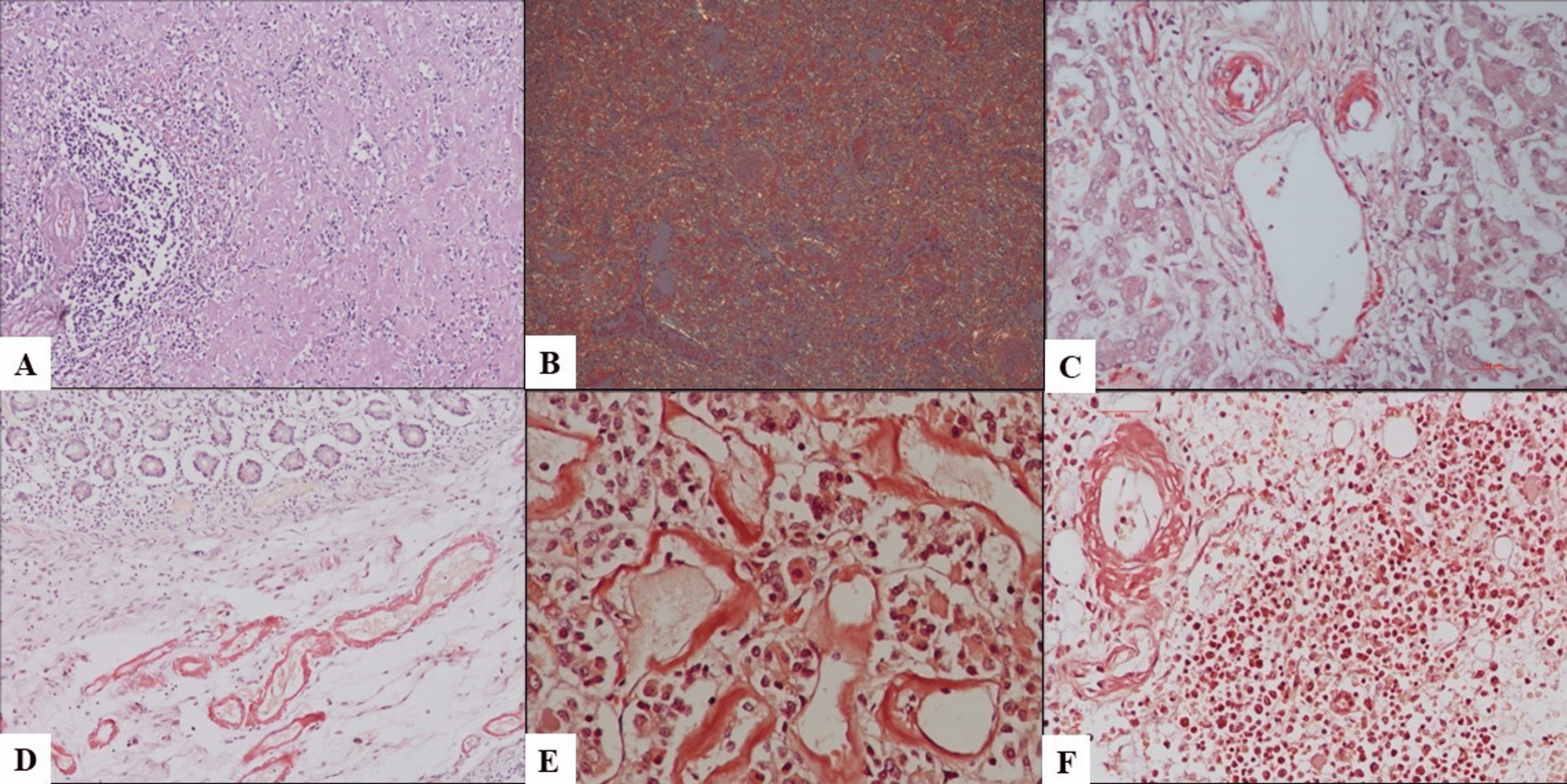
Demonstration of amyloidosis involving different organs. (A) Microscopy of spleen showing amyloid deposition within the sinusoids, sparing the periarteriolar lymphoid sheath, producing a lardaceous appearance (H&E, 100x). (B) Congo red stain demonstrates apple-green birefringence under polarized light (100x). (C)-(F) Similar congophilic amyloid deposits are observed in the hepatic artery, portal vein, small intestine, pituitary, and bone marrow (Congo red, 100x).

The bilateral kidneys weighed 450 g and were enlarged. The capsular surfaces were irregular and scarred. On sectioning, the cut surfaces were firm in consistency and exhibited a waxy feel. No focal lesions were identified. Microscopic examination revealed nearly all glomeruli to be enlarged, demonstrating marked mesangial expansion with obliteration of the capillary lumina due to deposition of pale, acellular, eosinophilic material. This material was weakly periodic acid-Schiff (PAS) positive, congophilic, and exhibited apple-green birefringence under polarized light, confirming amyloid deposition. Severe interstitial fibrosis and tubular atrophy (IFTA) were observed. No light chain restriction was detected on direct immunofluorescence. Immunohistochemistry for serum amyloid-associated (SAA) protein demonstrated intense positivity. Ultrastructural examination confirmed the presence of amyloid fibrils (Figures 1A-1F). Similar amyloid deposits were extensively noted in the sinusoids of the spleen, the hepatic artery, portal vein, and central venule of the liver, as well as the small arteries and arterioles of the small intestine, stomach, testes, pituitary gland, lymph nodes, and bone marrow (Figures 2A-2F).

**Figure 3:**
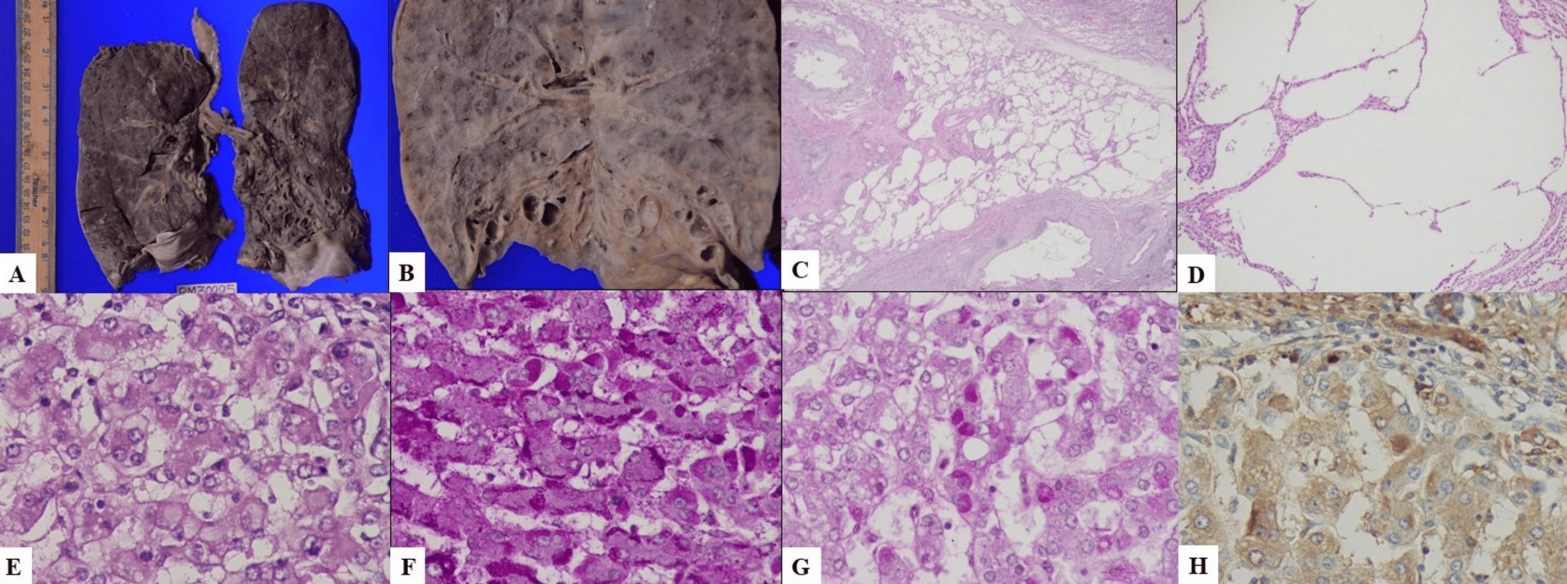
Gross and microphotographs of lung and liver. (A) Gross photograph of both lungs showing bronchiectasis. (B) Spongy emphysematous changes involving the lower lobes. (C) Microscopy reveals panacinar emphysema (H&E, 100x). (D) Higher magnification shows ruptured and floating interalveolar septa (H&E, 400x). (E) Microscopy of the liver showing ground-glass-like globules in periportal hepatocytes (H&E, 400x). (F) PAS stain highlights hyaline globules in periportal hepatocytes (PAS, 400x). (G) These globules are resistant to diastase digestion (PAS-D, 400x). (H) Immunostaining for alpha-1 antitrypsin shows strong positivity in the hyaline globules (DAB chromogen, 400x). PAS-D: periodic acid-Schiff with diastase, DAB: 3,3'-Diaminobenzidine.

Both lungs weighed 1100 g. The pleural surfaces showed fibrinous tags. On gross examination, the cut surfaces of both lungs were consolidated and exhibited grayish-white broncho-centric lesions. The trachea and major bronchi were grossly unremarkable. However, the segmental bronchi and bronchioles of the lower lobes were markedly dilated, reaching the pleural surfaces, and exhibited a globular ballooning appearance. Further slicing revealed spongy areas with cystic spaces (Figures 3A, 3B). Microscopic examination showed marked dilation of entire acini within lobules with thinned-out interalveolar septa, consistent with panacinar emphysema (Figures 3C, 3D). The terminal bronchioles and segmental bronchi were dilated, with bronchial walls showing mucus gland hyperplasia and lymphomononuclear inflammation. Sections from grayish-white nodules demonstrated bronchopneumonia with abscess formation. Special stains did not demonstrate any organisms, and polymerase chain reaction (PCR) assays for bacterial (16S rRNA) and fungal (18S rRNA) DNA were negative. Pre-acinar and inter-acinar arteries exhibited changes suggestive of pulmonary hypertension.

The liver weighed 1900 g and was enlarged with a greasy appearance. No focal lesions were identified. Microscopy revealed preserved lobular architecture and an intact reticulin framework. Masson trichrome staining did not highlight porto-porto or porto-central bridging fibrosis. Hepatocytes in the periportal regions showed microvesicular steatosis and cytoplasmic basophilia with globular appearances in some areas. PAS staining highlighted brightly PAS-positive hyaline globules within periportal hepatocytes, resistant to diastase digestion, consistent with alpha-1 antitrypsin deficiency (Figures 3E, 3F). Similar PAS-positive diastase-resistant hyaline globules were also seen focally in pneumocytes and alveolar macrophages within the lungs.

The heart chambers and valves were grossly unremarkable. Microscopy demonstrated similar PAS-positive diastase-resistant globules and proteinaceous material within myocardial fibers. The lymph nodes showed extensive hemophagocytosis.

Although genetic testing and serum alpha-1 antitrypsin levels were unavailable, the histopathological findings strongly suggested alpha-1 antitrypsin deficiency as the underlying cause of pulmonary disease and the secondary development of systemic SAA-associated amyloidosis.

## Discussion

Alpha-1 antitrypsin (AAT) deficiency is an inherited condition passed down in an autosomal codominant manner, meaning a person must inherit abnormal AAT genes from both parents to be affected. The condition is caused by changes in the SERPINA1 gene on chromosome 14, which has over 150 known variants. The most common and normal version is the M allele, and most people have two copies (MM), leading to normal AAT levels. Variants like the S and Z alleles reduce AAT levels, with the Z allele causing a more severe deficiency. People with ZZ are at high risk for AAT deficiency, while those with SZ have an increased risk of lung diseases such as emphysema, especially if they smoke [6,7]. Individuals with severe AAT deficiency often develop panacinar emphysema at a young age and may also present with chronic bronchitis and bronchiectasis [8]. Liver involvement stems from the polymerization of misfolded AAT within hepatocytes, leading to PAS-positive, diastase-resistant inclusions and, over time, hepatocellular injury [9]. While AAT deficiency is relatively common in individuals of European ancestry, it remains underdiagnosed in other populations, including those in India, due to low clinical suspicion and limited access to diagnostic testing [10].

In our case, the diagnosis of alpha-1 antitrypsin (AAT) deficiency was confirmed by the presence of classic histopathological features, including panacinar emphysema and periodic acid-Schiff with diastase (PAS-D)-positive hyaline globules in the liver, along with positive immunostaining for AAT. The chronic lung inflammation stemming from AAT deficiency likely led to sustained production of serum amyloid A protein, eventually culminating in systemic AA amyloidosis. This case highlights the intriguing interplay between two protein misfolding disorders: AAT deficiency and SAA-associated amyloidosis. While misfolded AAT accumulates intracellularly, aberrantly produced SAA circulates systemically, predisposing to amyloid fibril deposition across multiple organs [11]. Notably, despite extensive amyloid deposition, classical AAT deficiency-related liver dysfunction was not clinically evident in our patient, highlighting the heterogeneity of disease manifestations.

Additionally, the presence of extensive systemic amyloidosis with multiorgan involvement, including the kidneys, liver, gastrointestinal tract, lymph nodes, and lungs, underscores the severe burden of chronic inflammation. Importantly, this case highlights the potential for rare triggers, such as undiagnosed AAT deficiency, to drive systemic AA amyloidosis. Early recognition of AAT deficiency and intervention with therapies like augmentation therapy may prevent the long-term complications seen in this patient [12]. Furthermore, this case highlights the value of autopsy in uncovering hidden diagnoses and guiding future clinical vigilance for inherited conditions that predispose to systemic disease.

## Conclusions

While alpha-1 antitrypsin deficiency is more common in individuals of European descent, this deficiency is infrequently reported in India. Evaluation for AAT deficiency should be considered in cases of persistent dyspnea during childhood or adolescence, as well as when evaluating systemic SAA-associated amyloidosis. In suspected cases, AAT serum levels, isoelectric focusing for identifying AAT variants or genotyping of the protease inhibitor (PI) locus using polymerase chain reaction (PCR) or restriction fragment length polymorphisms can be done early for detection and prompt treatment.

Recognition and early diagnosis of AAT deficiency not only offer opportunities for targeted therapies but can also help prevent devastating complications like systemic amyloidosis. This case highlights the essential role of clinicopathological correlation and the irreplaceable value of autopsy in elucidating rare causes of systemic disease.
